# Emergence of pristinamycin resistance in India

**DOI:** 10.4103/0253-7613.48884

**Published:** 2009-02

**Authors:** Shyam Sunder Keshari, Arun Kumar Kapoor, Nira Kastury, Dharmendra Kumar Singh, Anudita Bhargava

**Affiliations:** 1 Department of Pharmacology and Microbiology, Moti Lal Nehru Medical College, Allahabad, Uttar Pradesh, India; 2 Department of Pediatrics, Sarojini Naidu Children Hospital, Allahabad, Uttar Pradesh, India

**Keywords:** Dalfopristin/quinupristin, pristinamycin resistance, staphylococcus

## Abstract

Quinupristin and dalfopristin combination has been advocated as a drug of choice for multi-drug resistant (MDR) gram-positive cocci (GPC). We are reporting two cases of neonatal septicemia, caused by the methicillin resistant *Staphylococcus aureus* (MRSA), showing primary *in vitro* pristinamycin resistance. The Minimum inhibitory concentrations (MIC) for pristinamycin in these two cases were 30 *μ*g and 25 *μ*g, respectively. Universal advocacy of pristinamycin for the therapy of MDR GPC infections should be re-evaluated.

## Introduction

Quinupristin and dalfopristin (SYNECID) is a combination of a streptogramin B, quinupristin, with a streptogramin A, dalfopristin, in a 30 : 70 ratio. These compounds are semisynthetic derivatives of naturally occurring pristinamycins, produced by *Streptomyces pristinaspiralis*. Quinupristin and dalfopristin are more soluble derivatives of pristinamycin IA and pristinamycin IIA respectively.[1] They bind with different targets in the peptidyltransferase domain of 23 ribosomal subunit, and inhibit protein elongation steps. Streptogramin A and B act synergistically *in vivo*; the mixture of the two components is bactericidal and the action is irreversible unlike their individual bacteriostatic activity.[2]

The first staphylococcal isolate resistant to the pristinamycin was reported in France, in 1975.[3] Staphylococcal resistance always pertains to dalfopristin, but not necessarily for quinupristin.[4] Quinupristin resistance to Staphylococci is mediated by various mechanisms like methylation of 23s rRNA,[5] inactivation of drug by lactonases,[1] mutation in the L22 ribosomal protein[6] and by efflux of the drug by ABC proteins.[7,8] Similarly, resistance to dalfopristin is mediated by plasmid genes like vat, vatB, vatC, vatD and satA;[4] staphylococcal genes vga, vgb, vgaB and lsa, which encode ATP-binding efflux proteins that pump type A compounds out of the bacterial cell.[4,8] A variant of vgaA (vgaAv) carried by a transposon (Tn5406) found in plasmid and/or chromosome of *staphylococci* was recently characterized.[9] We have isolated two clinical isolates of *Staphylococcus aureus*, which were resistant to pristinamycin (quinupristin/dalfopristin).

## Case Reports

### Case 1

A 17-day-old male baby was brought to the out-patient section of the pediatrics department, with a history of refusal to feed, sluggish activity and yellow discoloration of the body. There was no history of Rh-incompatibility. On physical examination, the neonate appeared ill and was icteric. His laboratory investigations were serum bilirubin - 17.6 mg%, serum calcium - 8.2 mg%, blood sugar (Random) - 72 mg%, haemoglobin-16.8 gm%, TLC - 5,800/cmm and DLC was polymorphs 82% and lymphocyte 18%. Blood culture was positive for MRSA. He was treated as a case of septicemia with Icterus neonatorum, in the neonatal intensive care unit (NICU).

### Case 2

A 5-day-old preterm female baby was brought to the out-patients section of the pediatric department with a history of refusal to feed, sluggish activity, yellow discoloration of the body, respiratory distress and seizure. There was no history of Rh-incompati-bility. On physical examination, the neonate appeared ill and icteric, with hypothermia, weak cry, tachycardia, tachypnea and poor motor activity. Her laboratory investigations were blood sugar (Random) - 112 mg%, serum bilirubin - 11.3 mg%, and serum calcium-3.9 mg%. Blood culture was positive for MRSA. She was treated in the NICU as a case of birth asphyxia with septicemia with Icterus neonatorum.

Blood culture and sensitivity was done as per standard protocol, in the Department of Microbiology, M.L.N. Medical College, Allahabad. The samples were collected in Brain heart infusion broth and periodically sub-cultured on MacConkey agar and blood agar. The colonies that grew were identified to the species level by a battery of biochemical tests. These isolates were tested by Kirby-Bauer disc diffusion method, against a panel of relevant antibiotics, using commercially available antibiotic discs.[10] The antibiotics tested included ampicillin, amoxycillin-clavulanic acid, amikacin, cephalexin, cephazolin, co-trimoxazole, clindamycin, ciprofloxacin, erythromycin, gentamycin, linezolid, oxacillin, pristinamycin (dalfopristin/quinupristin), tetracycline, teicoplanin and vancomycin. The MIC of pristinamycin was determined by E-test strips (Hicomb strips from Hi Media Laboratories, Pvt. Ltd., India).

## Result and Discussion

The blood samples of both the cases showed growth of MRSA. The MRSA was also resistant to pristinamycin by initial screening, using the Kirby Bauer disk diffusion method. The MICs for dalfopristin/quinupristin among these two isolates were 30 *μ*g ml^−1^ and 25 *μ*g ml^−1^. Although this test is not approved by the NCCLS, there are numerous studies which showed that E-test disk diffusion and broth micro dilution methods were comparable in accuracy for the susceptibility testing for MRSA and vancomycin resistant Enterococcus (VRE) against linezolid and quinupristin/dalfopristin.[11]

Earlier studies have shown very low MIC; MIC_90_ of 1-2 mg l^−1^ for MRSA and 0.5 -2 mg l^−1^ for CONS, *Streptococcus pneumoniae, Streptococcus viridans* and *Streptococcus pyogenes.* Earlier studies have reported that almost all isolates of MRSA and MRCONS are susceptible to dalfopristin/quinupristin.[11,12] However, our findings were in contrast to the earlier studies done in this field.

Infections due to gram-positive cocci are becoming more difficult to treat because of rapid emergence of antibiotic resistance and their dissemination in the population. Earlier observations have demonstrated that dalfopristin/quinupristin have good activity against MDR gram-positive cocci and are a promising therapeutic option in the era of rapidly increasing resistance in almost all parts of the world.

It is very interesting to find pristinamycin resistant *Staphylococcus aureus* in our country, where pristinamycin is not available *in vivo* for patient management. Pristinamycin is being used only as a research tool, by using antibiotic discs procured from a commercial source.

In the present study, the *Staphylococcal* isolates were found resistant to three or more antimicrobial agents among the panel of antibiotics tested. Therefore, they were defined as MDR *Staphylococcal* isolates. It may be postulated that resistance against pristinamycin is plasmid mediated. This plasmid may also contain genes conferring resistance against other commonly used antibiotics. Probably this gene was in a repressed state and was expressed on first exposure to the drug. Various genes have been identified which are responsible for causing streptogramin resistance, like erm, vgb A, vgb B, and msr genes.

More clinical isolates need to be tested against this antibiotic, so that the exact percentage of primary *in vitro* resistance can be known. Genetic studies for the responsible gene(s) should be done. After the above two cases, we are now storing all the pristinamycin resistant GPC isolates in our laboratory for further studies. We have isolated other pristinamycin resistant GPCs also, from specimens other than blood. After complete comprehensive studies on all such isolates have been done to find out the exact percentage of primary *in-vitro* resistance against this drug, in this part of our country. Thus, on the basis of our observation, it is advisable that more comprehensive region-wise laboratory work is done, before advocating this drug for empirical therapy for MDR GPC infections.
